# Person-Generated Health Data in Women’s Health: Protocol for a Scoping Review

**DOI:** 10.2196/26110

**Published:** 2021-05-28

**Authors:** Jalisa Lynn Karim, Aline Talhouk

**Affiliations:** 1 Department of Statistics and Actuarial Science University of Waterloo Waterloo, ON Canada; 2 Department of Obstetrics and Gynecology University of British Columbia Vancouver, BC Canada

**Keywords:** digital health, women’s health, mobile health, health app, wearables, femtech, self-tracking, personalized health, person-generated health data, patient-generated health data, scoping review

## Abstract

**Background:**

Due to their ability to collect person-generated health data, digital tools and connected health devices may hold great utility in disease prevention, chronic disease self-monitoring and self-tracking, as well as in tailoring information and educational content to fit individual needs. Facilitators and barriers to the use of digital health technologies vary across demographics, including sex. The “femtech” market is growing rapidly, and women are some of the largest adopters of digital health technologies.

**Objective:**

This paper aims to provide the background and methods for conducting a scoping review on the use of person-generated health data from connected devices in women’s health. The objectives of the scoping review are to identify the various contexts of digital technologies in women’s health and to consolidate women’s views on the usability and acceptability of the devices.

**Methods:**

Searches were conducted in the following databases: Medline, Embase, APA PsycInfo, CINAHL Complete, and Web of Science Core Collection. We included articles from January 2015 to February 2020. Screening of articles was done independently by at least two authors in two stages. Data charting is being conducted in duplicate. Results will be reported using the Preferred Reporting Items for Systematic Reviews and Meta-Analyses Extension for Scoping Reviews (PRISMA-ScR) checklist.

**Results:**

Our search identified 9102 articles after deduplication. As of November 2020, the full-text screening stage is almost complete and data charting is in progress. The scoping review is expected to be completed by Fall 2021.

**Conclusions:**

This scoping review will broadly map the literature regarding the contexts and acceptability of digital health tools for women. The results from this review will be useful in guiding future digital health and women’s health research.

**International Registered Report Identifier (IRRID):**

DERR1-10.2196/26110

## Introduction

### Background

Modern-day society is rapidly embracing innovations in connected technologies such as smartphones, text messages, wearables (eg, smartwatches), sensors, the Internet of Things (eg, internet-enabled weight scales) [1], as well as interactive web applications. When used in the context of health care, these technologies enable their users to collect, store, and reflect on their health data. Person-generated health data (PGHD) are defined as clinically relevant data captured outside traditional care settings [2] and describe experiences from the everyday lives of individuals. With information derived from this data, users are empowered to take actions toward improving their health [3-7].

The mass adoption of connected technologies and PGHD benefits person-centered care by enabling individuals to play a more active and proactive role in managing their health [8]. Connected health tools provide a platform for information exchange between individuals and their health care providers [9,10] in person and in telemedicine consultations. These tools can also be used asynchronously, at convenient times throughout the day, without requiring health care providers to be online simultaneously with the patient for a real-time conversation. Health outcomes can be measured and monitored weekly, daily, and continuously; these more frequent assessments provide users and health care providers quicker feedback on measures of health status, thus enabling faster medical interventions when necessary [11,12]. Detailed longitudinal PGHD can paint a more complete picture of one’s health, minimizing the risk of recall bias [13] at health appointments. Using PGHD to monitor health status over time can help detect health concerns early [14], prevent medical events [15,16], and evaluate patient outcomes during and after medical treatments [17,18].

One of the most promising features of digital health is the ability to personalize and tailor content to address specific health conditions and concerns. With or without consulting their medical team, participants can decide which health metrics are most pertinent to their situation and receive targeted information and feedback based on their personal measurements and symptoms [11,16]. Individualized health plans created from PGHD may encourage personal participation and accountability with respect to health and health-related behaviors [19,20]. The impersonal nature of these platforms, which provide the ability to ask questions and track their conditions anonymously, may be especially appreciated in scenarios where participants have reservations about discussing certain health issues in person with health care providers (eg, sexual health [21,22]); independently monitoring these health issues online allows them to be more forthcoming [23].

Applications specifically targeting women are exploding in what has been coined “femtech.” Historically, women were excluded from health research, which meant that very little was known about female-specific health concerns or diseases that impacted mostly women (eg, menstrual health) [24,25]. Considerable advancements have been made in women’s health over the past decades [26]. Today, health technology is promising to reverse the tide of research in women’s health with women 75% more likely to use digital health tools than men [27]. It is important that research in new areas such as digital health continues to recognize women’s needs and address their concerns, as sex and gender can influence adoption and acceptability of connected health technologies. 

Despite all the benefits of digital health technologies, many known barriers to successful adoption remain. Users of these technologies still have outstanding concerns around privacy and security of health data collected through these various devices. Data tracked by devices can contain large amounts of personal and sensitive information that users care to keep private [28] and to have more control over who can have access to such data [29-31]. The lack of perceived direct utility of digital health data [32,33] and the lack of applicable insight have slowed down the adoption of digital tools for clinical decision making [34,35]. Various studies have shown that the accuracy of wearable devices is variable and less reliable during dynamic activity outside of a laboratory [36,37]. The lack of adherence to using the technology over extended periods of time constitutes one of the biggest drawbacks from relying on such data. Users may forget or be unwilling to use the devices on a regular basis, and they may abandon self-tracking after a period of time if the perceived value is not realized [28]. Finally, rates of adoption of digital technologies vary greatly across sociocultural characteristics. Studies have found lower rates of adoption and more negative attitudes among individuals living in rural locations [38,39]. Younger individuals and women are more likely to use health apps and track health information online [40,41]. Women were less willing to share information, in comparison with men who were more confident about protecting their privacy [42,43]. Research has shown that individuals are more accepting of digital health technologies when the health information they deal with is less sensitive [31], and it is possible that women consider their female-specific health data (eg, menstruation, pregnancy) to be more sensitive than other types of general health data. As the femtech industry continues to grow, these concerns are becoming more prominent [44,45]. 

Scoping reviews on wearable technologies [46] and mobile health apps [47] have not addressed specific areas of women’s digital health. While some have looked at areas such as gestational diabetes [48], perinatal depression and anxiety [49], and fertility tracking [50], no study has broadly mapped the use of PGHD from connected devices for women’s health.

### Objectives

In this scoping review, we aim to explore the different contexts in which digital tools collecting PGHD are being proposed to address women’s health issues. We also want to evaluate women’s opinions with regards to the acceptability of connected health devices in these different contexts. More specifically, our review aims to answer the following research questions:

1. What are the different areas of women’s health or health-related behaviors that are being monitored with PGHD from connected health devices?
2. What personal metrics are being collected by these technologies?
3. What are the facilitators and barriers for women promoting or hindering their use of connected health devices?

The results from this review will allow us to identify gaps and unmet needs in women’s health research to help guide future digital and connected women’s health innovations.

## Methods

### Protocol Development

This scoping review protocol has been developed to align with the frameworks developed by Arksey and O’Malley [51] and Peters et al [52]. The completed Preferred Reporting Items for Systematic Reviews and Meta-Analyses Protocols (PRISMA-P) [53] checklist is provided in Multimedia Appendix 1.

### Search Strategy

The search strategy, developed in close collaboration with a reference librarian, was first created in Medline and adapted to Embase, APA PsycInfo, CINAHL Complete, and Web of Science Core Collection.

Initial searches were completed between March 2 and 6, 2020. Search alerts were used to include results added to the databases between March 6 and April 1, 2020. Manual searches were done to ensure that the initial search criteria used were comprehensive. On November 13, 2020, and March 10, 2021, we added additional search terms to broadly encompass possible missed articles. We kept a uniform cut-off date of February 29, 2020, for all included articles.

We focused on keywords and subject headings to ensure a broad coverage of the literature at the intersection of the following four topics: women, health, digital devices, and tracking. The topics of women and health were identified by terms such as “women’s health,” “female,” “mhealth,” and “digital health.” Terms such as “smartphone,” “wearable,” and “Internet of Things” were used to identify digital devices. Tracking was identified by terms including “tracking,” “monitoring,” “self-management,” “ResearchKit,” and “person-generated adj4 data.” The full list of search terms is included in Multimedia Appendix 2.

Terms referring to telemedicine (consultations with a health care provider in real time) were not included because we are primarily interested in technologies that allow the user to interact with the device on her own time for the collection of PGHD. Searches were limited to articles published in 2015 or later because publications with the keyword “digital health” started to emerge in the literature around that time [54,55]. We also excluded conference abstracts, conference reviews, editorials, letters, and comments due to limited feasibility and lack of details in such literature.

### Eligibility Criteria

Because we wished to broadly map the existing literature on digital data and connected women’s health research, we included a variety of studies: randomized and nonrandomized intervention studies, observational and correlative studies, feasibility and acceptability studies, case studies, reviews, descriptions of prototypes, measurement studies, analytical methods, and viewpoints. Study media releases and user reviews of specific applications were excluded. We only included articles written in English, irrespective of the country or the place of research.

We were interested in technologies and interventions targeting women, so papers were eligible if they specifically targeted women-only health topics (eg, pregnancy) or if they only included female participants. Articles including intersex, transgender, or nonbinary participants were not excluded.

We excluded articles that presented digital health tools designed for health care providers, as we are primarily interested in devices and apps that women can engage with independently outside of a clinical setting. Articles discussing the use of real-time consultations, whether through video, phone, or online chat, were excluded; however, some of the included interventions could involve the use of telemedicine services as long as they included asynchronous use. To maintain the focus of the review on tracking or monitoring one’s data for health, devices must have allowed users to input personal health data; therefore, publications reporting on apps or websites used solely for educational purposes were excluded. Complete inclusion and exclusion criteria are presented in Textboxes 1 and 2.

**Inclusion criteria.**

- Published between January 1, 2015, and February 29, 2020
- Refers to a health issue that pertains only to women or consists of only female participants of any age
- Includes the use of connected health tools for tracking or monitoring some aspect of health. This could include smartphones, wearable devices, the Internet of Things (eg, Bluetooth- or internet-enabled glucometers, blood pressure cuffs, and weight scales), and implantable devices
- Involves data collection from the user of the connected health tool (ie, the user either manually inputs data into the device or it is automatically uploaded)
- The user must be able to interact with the app or device on her own at home (outside of a clinical setting)
- Available in English

**Exclusion criteria.**

- Not available in English
- Conference abstracts, conference reviews, editorials, letters, or comments
- Study media releases and user reviews of specific applications
- Research conducted on animals
- Research involving male participants
- Tracking of infants and children, with the exception of tracking breastfeeding (since breastfeeding is directly related to the mother’s health and body)
- Devices or apps that are meant for health care provider use, use in a clinical setting only, or cannot be used independently without a health care provider present
- Digital health tools that are only for educational or informational purposes and do not allow the user to enter or track her own data (ie, no information exchange)
- Telemedicine services (eg, live video consultations with health care providers)

### Study Selection

Results from the database searches were imported to the Covidence systematic review software [56] and deduplicated. Screening of articles occurred in two stages. First, titles and abstracts were independently screened by at least 2 reviewers according to the eligibility criteria defined above. For articles meeting the inclusion and exclusion criteria at the title and abstract level, the full texts were then reviewed independently, also by 2 different reviewers. Conflicts at either stage were discussed and agreed upon between members of the research team.

If we were unable to locate the full text of the article online or through the library, we made a request to the corresponding authors. Articles that remained inaccessible were excluded at this stage, and the count of such articles will be reported in the PRISMA flow diagram in the completed review.

### Data Charting

For each article included, data will be charted by 1 reviewer in a spreadsheet and verified for accuracy by a second reviewer. A preliminary list of data charting elements was proposed and finalized (Table 1) after charting data from a dozen articles. The research team discussed which elements provided the most useful information and which to discontinue, and added important components of the papers if they were not being adequately captured by our preliminary list. 

To investigate the different contexts of women’s health that use PGHD from connected health devices, we will record all health area(s) of focus for each article. These areas refer to categories such as maternal health and fetal monitoring, menstruation, gestational diabetes, physical activity, etc. We will also record the year of publication and the country in which the research was conducted. To better understand which health metrics are most collected, we will document the types of connected health technologies discussed in the different studies (eg, wearable), the name of the devices or apps if applicable (eg, Fitbit Charge 2), and the personal metrics collected by the technologies (eg, daily step counts). Finally, to answer the third research question about facilitators and barriers, we will record any comments about the usability and acceptability of the technologies, including features that users liked or disliked. We will not focus on the outcome results of intervention studies, as that would be outside the scope of our review. 

**Table 1:** Data charting elements.

Type of data	Details of charted data
Article information	Title Authors Year of first publication
Study characteristics	Country in which the research was conducted Research study design
Contexts for women’s connected health	Health areas of focus (eg, pregnancy, obesity, diabetes, etc)
Digital device details	Types of digital health (eg, smartphone app, wearable, Internet of Things, implantable device, etc) Name of device or app Metrics collected by the devices (eg, heart rate, temperature, daily steps, pain rating, etc)
Usability and acceptability	Facilitators to use of the technologies Barriers to use of the technologies

### Presentation of Results

A PRISMA flow diagram will be presented to detail the study selection process. Tables and graphs will be used to report findings on the contexts of the applications, including the various health areas of focus and metrics collected. A thematic analysis will be conducted to identify categories of facilitators and barriers in discussions about the acceptability and usability of the digital health technologies. Exact details of the format of the report will be determined as a team after completing data charting.

## Results

The searches from March 2020 returned 11,533 results, and the additional searches run in November 2020 and March 2021 returned 3096 results. There were a total of 9102 articles to screen after deduplication in Covidence. We did not encounter any articles in our search that mentioned the inclusion of intersex, transgender, or nonbinary participants. As of November 2020, the full-text screening stage is nearly complete and reviewers have started data charting. Results are expected to be submitted for publication by Fall 2021 and reported in accordance with the PRISMA Extension for Scoping Reviews (PRISMA-ScR) guidelines [57].

## Discussion

Reviews have previously been conducted in specific areas of women’s health and digital health. However, to the authors’ knowledge, no review has been conducted with the broad scope proposed here. This scoping review will provide an extensive report of the literature regarding connected digital health devices for monitoring in all areas of women’s health through the collection of PGHD. This information will be useful for women’s health and digital health researchers to identify new research questions and design their programs according to the identified facilitators and barriers to use.

One limitation of our scoping review is that it does not include conference abstracts, conference reviews, editorials, letters, comments, or gray literature. Although we are including research conducted worldwide, our review does not include non-English articles. Finally, due to the nature of scoping reviews, quality assessments will not be performed on included articles. However, we are not assessing the outcome results of intervention studies, so this is not pertinent to our review.

## Appendix

Multimedia Appendix 1 PRISMA-P checklist.

Multimedia Appendix 2 Electronic search strategy.

## Abbreviations

PGHD: person-generated health data
PRISMA-P: Preferred Reporting Items for Systematic Reviews and Meta-Analyses Protocols
PRISMA-ScR: PRISMA Extension for Scoping Reviews
