# Correction: Person-Generated Health Data in Women’s Health: Protocol for a Scoping Review

**DOI:** 10.2196/34211

**Published:** 2021-10-18

**Authors:** Jalisa Lynn Karim, Aline Talhouk

**Affiliations:** 1 Department of Statistics and Actuarial Science University of Waterloo Waterloo, ON Canada; 2 Department of Obstetrics and Gynecology University of British Columbia Vancouver, BC Canada

In “Person-Generated Health Data in Women’s Health: Protocol for a Scoping Review” (JMIR Res Protoc 2021;10(5):e26110), one error was noted.

The phone number for the Corresponding Author in the originally published manuscript has been removed and replaced by the phone number “1 604 875 4111.”

The correction will appear in the online version of the paper on the JMIR Publications website on October 18, 2021, together with the publication of this correction notice. Because this was made after submission to PubMed, PubMed Central, and other full-text repositories, the corrected article has also been resubmitted to those repositories.

