# Supplementary material for: Person-Generated Health Data in Women’s Health: Protocol for a Scoping Review
Source: JMIR Res Protoc. 2021 May 28;10(5):e26110. doi: 10.2196/26110 (PMC8196349; doi:10.2196/26110)
Supplement: Multimedia Appendix 2 [file resprot_v10i5e26110_app2.pdf]

This multimedia appendix contains the full search strategy used in our scoping review titled “Person-Generated Health Data in Women’s Health”.

Terms added for the additional searches run on November 13 2020 and March 10 2021 are highlighted in grey. These terms were not originally included in the March 2020 searches.

Ovid MEDLINE(R) and Epub Ahead of Print, In-Process & Other Non-Indexed Citations and Daily <1946 to March 02, 2020>

#	Searches
1	medical informatics applications/
2	mobile applications/
3	exp Computers, Handheld/
4	exp Cell Phone/
5	Wireless Technology/
6	exp Wearable Electronic Devices/
7	((mobile or smart* or phone? or cellular or cellphone? or tablet?) adj4 (App? or application? or software)).kw,tw.
8	((mobile or smart* or tablet? or wireless or hand-held or handheld or portable) adj4 (device? or technolog*)).kw,tw.
9	(medical informatics or health informatics or mobile health or mhealth or m-health or electronic health or ehealth or e-health or digital health or smartphone? or smartwatch* or tablet computer? or electronic tablet? or electronic device? or wireless communication? or ipad? or i-pad? or iphone? or i-phone? or android or wearable? or Internet of things or IOT).kf,tw.
10	or/1-9
11	Monitoring, Physiologic/
12	Self Care/
13	Self-management/
14	(("person-generated" or "patient-generated") adj4 data).kw,tw.
15	(“digital biomark*” or track* or monitor* or sensor* or nanosensor? or self-care or self-manag* or self-regulat* or PGHD or ResearchKit or ResearchStack or HealthKit or CareKit or "Google Fit" or "Apple Health").kf,tw.
16	or/11-15
17	exp Women's health/
18	exp Women/
19	Female/
20	(wom#n or women? or female?).kf,tw.
21	or/17-20
22	10 and 16 and 21
23	22 not (Animals/ not (Animals/ and Humans/))
24	(comment or editorial or letter).pt.
25	23 not 24
26	limit 25 to yr="2015 - 2020"

Embase <1974 to 2020 March 02>

#	Searches
1	medical informatics/
2	mobile application/
3	mobile health application/
4	tablet computer/
5	exp wearable computer/
6	exp mobile phone/
7	wireless communication/
8	electronic device/
9	activity tracker/
10	((mobile or smart* or phone? or cellular or cellphone? or tablet?) adj4 (App? or application? or software)).ab,kw,ti.
11	((mobile or smart* or tablet? or wireless or hand-held or handheld or portable) adj4 (device? or technolog*)).ab,kw,ti.
12	(medical informatics or health informatics or mobile health or mhealth or m-health or electronic health or ehealth or e-health or digital health or smartphone? or smartwatch* or tablet computer? or electronic tablet? or electronic device? or wireless communication? or ipad? or i-pad? or iphone? or i-phone? or android or wearable? or Internet of things or IOT).ab,kw,ti.
13	or/1-12
14	home monitoring/
15	physiologic monitoring/
16	self monitoring/
17	patient monitoring/
18	personal monitoring/
19	online monitoring/
20	sensor/
21	electronic sensor/
22	nanosensor/
23	self care/
24	(("person-generated" or "patient-generated") adj4 data).ab,kw,ti.
25	(“digital biomark*” or track* or monitor* or sensor* or nanosensor? or self-care or self-manag* or self-regulat* or PGHD or ResearchKit or ResearchStack or HealthKit or CareKit or "Google Fit" or "Apple Health").ab,kw,ti.
26	or/14-25
27	women's health/
28	female/
29	(wom#n or women? or female?).ab,kw,ti.
30	or/27-29
31	13 and 26 and 30
32	31 not (Animals/ not (Animals/ and Humans/))
33	(conference abstract or conference review or editorial or letter).pt.
34	32 not 33
35	limit 34 to yr="2015 - 2020"

APA PsycInfo
Search completed: March 4, 2020

#	Searches
S1	DE "Mobile Health"
S2	DE "Mobile Applications"
S3	DE "Tablet Computers"
S4	DE "Mobile Phones"
S5	DE "Wireless Technologies"
S6	DE "Mobile Technology"
S7	DE "Mobile Devices"
S8	DE "Wearable Devices"
S9	TI ((mobile OR smart* OR phone# OR cellular OR cellphone# OR tablet#) N3 (App# OR application# OR software)) OR AB ((mobile OR smart* OR phone# OR cellular OR cellphone# OR tablet#) N3 (App# OR application# OR software)) OR KW ((mobile OR smart* OR phone# OR cellular OR cellphone# OR tablet#) N3 (App# OR application# OR software))
S10	TI ((mobile OR smart* OR tablet# OR wireless OR hand-held OR handheld OR portable) N3 (device# OR technolog*)) OR AB ((mobile OR smart* OR tablet# OR wireless OR hand-held OR handheld OR portable) N3 (device# OR technolog*)) OR KW ((mobile OR smart* OR tablet# OR wireless OR hand-held OR handheld OR portable) N3 (device# OR technolog*))
S11	TI ("medical informatics" OR "health informatics" OR "mobile health" OR mhealth OR m-health OR "electronic health" OR ehealth OR e-health OR "digital health" OR smartphone# OR smartwatch* OR "tablet computer#" OR "electronic tablet#" OR "electronic device#" OR "wireless communication#" OR ipad# OR i-pad# OR iphone# OR i-phone# OR android OR wearable# OR "internet of things" OR iot) OR AB ("medical informatics" OR "health informatics" OR "mobile health" OR mhealth OR m-health OR "electronic health" OR ehealth OR e-health OR "digital health" OR smartphone# OR smartwatch* OR "tablet computer#" OR "electronic tablet#" OR "electronic device#" OR "wireless communication#" OR ipad# OR i-pad# OR iphone# OR i-phone# OR android OR wearable# OR "internet of things" OR iot) OR KW ("medical informatics" OR "health informatics" OR "mobile health" OR mhealth OR m-health OR "electronic health" OR ehealth OR e-health OR "digital health" OR smartphone# OR smartwatch* OR "tablet computer#" OR "electronic tablet#" OR "electronic device#" OR "wireless communication#" OR ipad# OR i-pad# OR iphone# OR i-phone# OR android OR wearable# OR "internet of things" OR iot))
S12	S1 OR S2 OR S3 OR S4 OR S5 OR S6 OR S7 OR S8 OR S9 OR S10 OR S11
S13	DE "Monitoring"
S14	DE "Self-Monitoring"
S15	DE “Tracking”
S16	DE “Self-Care”
S17	DE “Self-Management”
S18	DE “Self-Regulation”
S19	TI (("person-generated" OR "patient-generated") N3 data) OR AB (("person-generated" OR "patient-generated") N3 data) OR KW (("person-generated" OR "patient-generated") N3 data)
S20	TI (“digital biomark*” OR track* OR monitor* OR sensor* OR nanosensor# OR self-care OR self-manag* OR self-regulat* OR PGHD OR ResearchKit OR ResearchStack OR

	HealthKit OR CareKit OR "Google Fit" OR "Apple Health") OR AB ("digital biomark*" OR track* OR monitor* OR sensor* OR nanosensor# OR self-care OR self-manag* OR self-regulat* OR PGHD OR ResearchKit OR ResearchStack OR HealthKit OR CareKit OR "Google Fit" OR "Apple Health") OR KW ("digital biomark*" OR track* OR monitor* OR sensor* OR nanosensor# OR self-care OR self-manag* OR self-regulat* OR PGHD OR ResearchKit OR ResearchStack OR HealthKit OR CareKit OR "Google Fit" OR "Apple Health")
S21	S13 OR S14 OR S15 OR S16 OR S17 OR S18 OR S19 OR S20
S22	DE "Human Females"
S23	TI (wom?n OR women# OR female#) OR AB (wom?n OR women# OR female#) OR KW (wom?n OR women# OR female#)
S24	S22 OR S23
S25	DE "Health" OR DE "Adolescent Health" OR DE "Health Anxiety" OR DE "Health Status" OR DE "Global Health" OR DE "Health Disparities" OR DE "Health Literacy" OR DE "Health Outcomes" OR DE "Health Promotion" OR DE "Holistic Health" OR DE "Mental Health" OR DE "Occupational Health" OR DE "Oral Health" OR DE "Physical Health" OR DE "Population Health" OR DE "Public Health" OR DE "Reproductive Health" OR DE "Sexual Health" OR DE "Well Being"
S26	TI health OR AB health OR KW health
S27	S25 OR S26
S28	S12 AND S21 AND S24 AND S27
S29	S28 NOT (DE "Animals" OR DE "Species Differences" OR DE "Animal Limb" OR DE "Animal Offspring" OR DE "Female Animals" OR DE "Infants (Animal)" OR DE "Invertebrates" OR DE "Male Animals" OR DE "Pets" OR DE "Service Animals" OR DE "Vertebrates") NOT ((DE "Animals" OR DE "Species Differences" OR DE "Animal Limb" OR DE "Animal Offspring" OR DE "Female Animals" OR DE "Infants (Animal)" OR DE "Invertebrates" OR DE "Male Animals" OR DE "Pets" OR DE "Service Animals" OR DE "Vertebrates") AND DE "Human Females")
S30	S29 NOT (ZZ "editorial" or ZZ "letter" or ZZ "comment/reply")
Limited by: Publication Year: 2015-2020	

CINAHL Complete
Search completed: March 6, 2020

#	Searches
S1	MH "Health Informatics"
S2	MH "Medical Informatics"
S3	MH "Mobile Applications"
S4	MH "Computers, Portable+"
S5	MH "Cellular Phone"
S6	MH "Wireless Communications"
S7	MH "Fitness Trackers"
S8	TI ((mobile OR smart* OR phone# OR cellular OR cellphone# OR tablet#) N3 (App# OR Application# OR Software)) OR AB ((mobile OR smart* OR phone# OR cellular OR cellphone# OR tablet#) N3 (App# OR Application# OR Software))
S9	TI ((mobile OR smart* OR tablet# OR wireless OR hand-held OR handheld OR portable) N3 (device# OR technolog*)) OR AB ((mobile OR smart* OR tablet# OR wireless OR hand-held OR handheld OR portable) N3 (device# OR technolog*))

S10	TI ("medical informatics" OR "health informatics" OR "mobile health" OR mhealth OR m-health OR "electronic health" OR ehealth OR e-health OR "digital health" OR smartphone# OR smartwatch* OR "tablet computer#" OR "electronic tablet#" OR "electronic device#" OR "wireless communication#" OR ipad# OR i-pad# OR iphone# OR i-phone# OR android OR wearable# OR "internet of things" OR iot) OR AB ("medical informatics" OR "health informatics" OR "mobile health" OR mhealth OR m-health OR "electronic health" OR ehealth OR e-health OR "digital health" OR smartphone# OR smartwatch* OR "tablet computer#" OR "electronic tablet#" OR "electronic device#" OR "wireless communication#" OR ipad# OR i-pad# OR iphone# OR i-phone# OR android OR wearable# OR "internet of things" OR iot)
S11	S1 OR S2 OR S3 OR S4 OR S5 OR S6 OR S7 OR S8 OR S9 OR S10
S12	MH "Monitoring, Physiologic"
S13	MH "Wearable Sensors+"
S14	MH “Self Care”
S15	MH “Self-Management”
S16	MH “Blood Glucose Self-Monitoring”
S17	MH “Self Regulation”
S18	TI (("person-generated" OR "patient-generated") N3 data) OR AB (("person-generated" OR "patient-generated") N3 data)
S19	TI (“digital biomark*” OR track* OR monitor* OR sensor* OR nanosensor# OR self-care OR self-manag* OR self-regulat* OR PGHD OR ResearchKit OR ResearchStack OR HealthKit OR CareKit OR "Google Fit" OR "Apple Health") OR AB (“digital biomark*” OR track* OR monitor* OR sensor* OR nanosensor# OR self-care OR self-manag* OR self-regulat* OR PGHD OR ResearchKit OR ResearchStack OR HealthKit OR CareKit OR "Google Fit" OR "Apple Health")
S20	S12 OR S13 OR S14 OR S15 OR S16 OR S17 OR S18 OR S19
S21	MH "Women's Health"
S22	MH "Women+"
S23	MH "Female"
S24	TI (wom?n OR women# OR female#) OR AB (wom?n OR women# OR female#)
S25	S21 OR S22 OR S23 OR S24
S26	S11 AND S20 AND S25
S27	S26 NOT (MH "Animals+" NOT (MH "Animals+" AND MH "Human"))
S28	S27 NOT (PT commentary OR editorial OR letter OR "letter to the editor")
Limited by: Publication Year: 2015-2020	

Web of Science Core Collection
Search completed: March 3, 2020

#	Searches
#1	TS =(“medical informatics” or “health informatics” or “mobile health” or mhealth or m-health or “electronic health” or ehealth or e-health or “digital health” OR smartphone$ or smartwatch* or “tablet computer$” or “electronic tablet$” or “electronic device$” or “wireless communication$” or ipad$ or i-pad$ or iphone$ or i-phone$ or android or wearable$ or “internet of things” or iot)
#2	TS =((mobile OR smart* OR phone$ OR cellular OR cellphone$ OR tablet$) near/3 (app$ OR application OR software))

#3	TS =((mobile OR smart* OR tablet$ OR wireless OR hand-held OR handheld OR portable) near/3 (device$ OR technolog*))
#4	#1 OR #2 OR #3
#5	TS=(“digital biomark*” OR track* OR monitor* OR sensor* OR nanosensor$ OR self-care OR self-manag* OR self-regulat* OR PGHD OR ResearchKit OR ResearchStack OR HealthKit OR CareKit OR “Google Fit” OR “Apple Health”)
#6	TS=(("person-generated" OR "patient-generated") near/3 data)
#7	#5 OR #6
#8	TS = (Wom?n OR women$ OR female$)
#9	TS = health
#10	#4 AND #7 AND #8 AND #9
**Refined By:** [excluding] **DOCUMENT TYPES:** (EDITORIAL MATERIAL) AND **PUBLICATION YEARS:** (2020 OR 2019 OR 2018 OR 2017 OR 2016 OR 2015)	
